# Hybrid Attention Network for Language-Based Person Search

**DOI:** 10.3390/s20185279

**Published:** 2020-09-15

**Authors:** Yang Li, Huahu Xu, Junsheng Xiao

**Affiliations:** 1 School of Computer Engineering and Science, Shanghai University, Shanghai 200444, China; jsxiao@shu.edu.cn; 2 School of Information Technology, Shanghai Jianqiao University, Shanghai 201306, China

**Keywords:** language-based person search, cross-modal retrieval, hybrid attention, joint learning

## Abstract

Language-based person search retrieves images of a target person using natural language description and is a challenging fine-grained cross-modal retrieval task. A novel hybrid attention network is proposed for the task. The network includes the following three aspects: First, a cubic attention mechanism for person image, which combines cross-layer spatial attention and channel attention. It can fully excavate both important midlevel details and key high-level semantics to obtain better discriminative fine-grained feature representation of a person image. Second, a text attention network for language description, which is based on bidirectional LSTM (BiLSTM) and self-attention mechanism. It can better learn the bidirectional semantic dependency and capture the key words of sentences, so as to extract the context information and key semantic features of the language description more effectively and accurately. Third, a cross-modal attention mechanism and a joint loss function for cross-modal learning, which can pay more attention to the relevant parts between text and image features. It can better exploit both the cross-modal and intra-modal correlation and can better solve the problem of cross-modal heterogeneity. Extensive experiments have been conducted on the CUHK-PEDES dataset. Our approach obtains higher performance than state-of-the-art approaches, demonstrating the advantage of the approach we propose.

## 1. Introduction

In today’s society, video surveillance has become an important means of public security, and thousands of surveillance cameras have been installed in public places. It is a very difficult task to search for a person manually in large-scale video data, as it consumes too much manpower and time and reduces the efficiency of police handling cases. In recent years, the automatic search of interested persons in large-scale video or image database has attracted the increasing attention of researchers. There are two main ways to conduct a person search: Image/video-based and text-based.

Image/video-based person search (IBPS) refers to the use of computer vision (CV) techniques to determine whether the specified person in the camera appears in other cameras, also known as person re-identification (reID) [1,2]. In recent years, person reID has attracted extensive interest and become a hot spot of CV. After more than 10 years of development, person reID technology has achieved high accuracy on the public person reID datasets. However, person reID has great limitations, because it requires that at least one image of the target person can be obtained, but in some actual cases, it may not be able to obtain the image of the target person. In this case, the target person can only be searched in the surveillance video/image database based on the text language description of the target person’s appearance provided by the witness, which is called text-based person search (TBPS).

**Figure 1:**
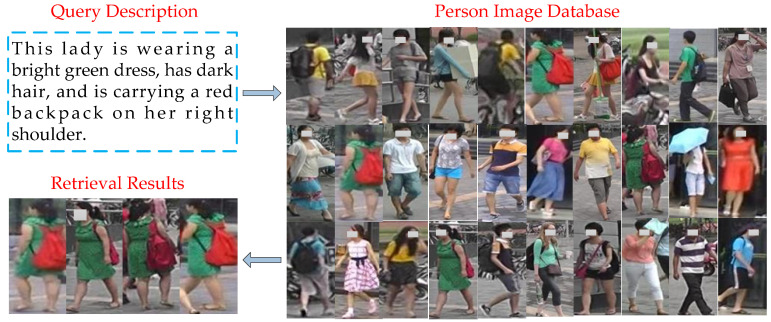
A graphical representation of a natural language-based person search. Provided a language description about the target person, the method retrieves the corresponding person images from the person image database.

TBPS can be classified into two classes: Attribute and natural language. Attribute-based person search involves searching for a person with certain attributes in the database [3,4,5], such as wearing a red t-shirt, etc. This method needs a set of predefined semantic attributes to describe the appearance of person, and the ability of attributes to describe the appearance of person is limited. It is also very expensive to mark attributes for large-scale person image datasets. Because of the low-dimensional feature of person attributes, the results of attribute-based person search are often not satisfactory. A person search based on natural language refers to searching the closest person image in large-scale video surveillance dataset through a language description given by the witness [6,7,8], as shown in Figure 1. The description of witness is often a natural language description rather than discrete attributes. Natural language is able to represent the appearance details of person more accurately than the attributes, and can provide more semantic information for person search. Therefore, this paper studies person search tasks based on natural language query description.

A person search based on natural language involves using natural language description to retrieve person images in large-scale datasets, which is actually a fine-grained application field of cross-modal retrieval. It needs to deal with both CV and natural language processing (NLP) at the same time, which has great technical difficulties and challenges. On the one hand, because of the modal heterogeneity, it is difficult to directly measure the cross-modal similarity and correlation between the language description features and the image appearance features. On the other hand, because all images are part of the same big class (that is, person class), the discriminations among different persons are not obvious, which is a typical fine-grained recognition problem. Also, different witnesses may describe the same person differently, which further increases the difficulty of the task. In short, when dealing with the task, there are two main difficulties: Fine-grained and cross-modality. This paper focuses on the solution of these two challenging difficulties.

For the challenge of fine-grained, one key method is to enhance the ability of discriminative feature extraction and to extract the discriminative feature representation associated with language description and visual appearance. We discuss feature extraction for image and text, respectively.

To enhance the ability of discriminative feature extraction for image, this paper mainly focuses on two aspects: Introducing an attention mechanism and leveraging midlevel features. As we know, attention mechanism plays an important role in the human visual perception system [9]. Human visual perception does not process the whole image at one time but instead continuously glimpses and selectively focuses on the critical parts to obtain key visual information [10]. This feature is conducive to the learning of discriminant features of the image. For a person search, local features play an important role in distinguishing different persons. Local features are learned from different locations of the person image, which corresponds to spatial attention (SA). Spatial attention mainly focuses on “where” the critical parts of person images are. In addition, in the convolution neutral network (CNN), different features are dispersed in different channels of the feature map. The network usually uses the full connection layer and softmax loss at the highest layer for classification tasks. Every channel of the feature map at the highest layer of the network can be regarded as a response of each class, and different semantic class responses are interrelated [11]. In the person search task, different classes correspond to different person IDs. Therefore, by exploring the relationship among different channels in the feature map, person features can be enhanced to have specific body semantic information, which corresponds to channel attention (CA). CA mainly focuses on “what” the critical parts of person images are. Both SA and CA are used is this paper and combined in an improved way to produce the novel cubic attention mechanism that we proposed.

The features extracted from different layers of CNN have different characteristics and values. Features of the lower-level network have more details and more spatial information, which can provide more valuable information for distinguishing different persons. It is very beneficial to the fine-grained task of person search. Most of the existing methods only extract high-level semantic features [6,7]. The high-level network has rich semantic features, but the extracted information is too abstract, leading to the problem of insufficient detail information, which affects the person search performance. Different from most other methods just generating both spatial attention and channel attention based on the highest convolution layer or same layer of the network [12], we proposed a cross-layer cubic attention mechanism which generates spatial attention based on the midlevel network and generates channel attention based on the high-level network, to fully leverage the spatial information and rich details of the midlevel network and rich semantics of the high-level network, so as to get better performance of this fine-grained task. The reason we did not generate spatial attention based on the low-level network is because of the insufficient semantic information and high computational cost of the low-level network.

To enhance the ability of discriminative feature extraction for language description, we proposed text attention network based on BiLSTM [13,14] and the self-attention [15,16] mechanism. The language descriptions of persons are context-dependent, and the contribution of each word to semantics is different. Through the forward and backward propagation of BiLSTM, it can better capture the hidden dependency of context and obtain the features with richer semantics. In most of the existing methods [7], the output vectors of BiLSTM at every time step are summed and averaged directly, enabling each word to have the same contribution to semantics, which easily leads to unsatisfactory performance. In this work, self-attention was used to fully consider the contribution of different words to semantics, to pay more attention to the key words in language description, to increase the weight of key words, and to reduce the weight of redundant or unimportant words, so as to obtain more discriminative feature representation of language description and improve the performance of the task.

To deal with the challenge of cross-modality, we proposed a cross-modal attention mechanism and joint loss function. Most of the existing works have independently extracted the features of each modality, and then measured the cross-modal correlation and similarity of the features [7,17]. However, the features of different modalities are quite different and noisy, and the correlation between features is not strong because of cross-modal heterogeneity. This paper proposes a cross-modal attention mechanism (CMAM) to solve the problem. The CMAM is able to fully acquire the correlation between text and image feature and focuses on the correlated parts between features. Thereby, it improves the final performance of this cross-modal task.

A joint loss function for cross-modal learning is designed in this paper. First, ranking loss was used because it has a good effect in cross-modal retrieval. However, the commonly used ranking loss only requires the network to distinguish the person image based on whether the image matches the language description or not (positive image-text sample pair or negative image-text sample pair). This constraint is coarse-grained. The ranking loss is improved for the task, and a new ranking loss embedded with intra-modal similarity is proposed. The sample distance in the same modality was calculated as the coefficient of the negative sample pair distance in the new similarity ranking loss, which can not only distinguish the positive pair and negative pair in coarse-grained, but also distinguish the similarity between the negative pairs in fine-grained way, so as to achieve a more accurate language-based person search ranking. Second, the similarity ranking loss mainly concerns cross-modal correlation and cannot well explore the intra-modal distribution of samples. For this reason, classification loss was also used, which uses the identity label information to mine intra-modal semantic information. Classification loss was also leveraged to constrain similarity ranking loss based on the identity label, which enabled the samples of the same identity to be aggregated as much as possible, so as to obtain more distinctive features to further increase the accuracy of the person search. Finally, similarity ranking loss and classification loss were joined to form a joint loss function for cross-modal learning and jointly train the network.

The major contributions of our work are listed below:

- A cross-layer cubic attention mechanism was proposed to enhance the ability of discriminative feature extraction for image. It can not only focus on critical features, but also fully leverages both the detailed features of midlevel network and the semantic features of high-level network, so that the performance of this cross modal fine-grained person search task can be improved.
- A text attention network, including both BiLSTM and self-attention, was put forward to enhance the ability of discriminative feature extraction for language description by better capturing the hidden dependency of context and increasing the weight of key words.
- A cross-modal attention mechanism was proposed, which can pay more attention to the correlated important parts between text and image features and better solve the problem of cross-modal heterogeneity.
- A joint loss function for cross-modal learning was proposed, including improved cross-modal ranking loss embedded with intra-modal similarity and intra-modal classification loss. The loss function leverages similarity ranking and discriminant feature mining to further improve the performance of natural language description based person search.

## 2. Related Works

In the section, existing studies on person search are briefly reviewed. The works can be classified into following two classes: Image/video-based person search (IBPS) and text-based person search (TBPS).

### 2.1. Image/Video-Based Person Search

Deep learning approaches use deep neural network to automatically acquire the feature representation of persons and utilize deep nonlinear functions to mine the representation distribution of the datasets, which have achieved remarkable success in the task of person searches based on image/video. Generally speaking, researchers mainly use three kinds of network frameworks to learn the features of person: Verification network, classification network, and ranking network frameworks. Based on these three frameworks, different network structures and loss functions have been designed to improve person search performance. Varior et al. adopted a long-short term memory (LSTM) network to process image regions in sequence after image segmentation. The method can use context information to increase the discrimination ability of local features [18]. Liu et al. applied an attention mechanism to multiple feature layers, which fused those salient features extracted in multiple layers, and enabled the extracted features to contain not only the detailed appearance features, but also the semantic features [19]. He et al. added a reconstruction module to the network to reconstruct the query image with candidate images, which can match the nonaligned images of different sizes and different locations [20]. Song et al. used the body foreground binary mask to guide the network attention to select the features of foreground region and background region, respectively [21]. Guo et al. used ranking loss and classification loss to match constraints under multiple feature scales and found that using small networks can also help to obtain much better search results [22]. In view of the fact that it is easy to obtain person data and difficult to obtain person labels in practical applications, researchers have tried to combine deep learning with semi-supervised learning algorithm [23] or unsupervised learning algorithm [24], and learn person features on unlabeled data through feature clustering and network fine-tuning strategy. Both semi-supervised and unsupervised algorithms have been often used in cross-domain person reID tasks for the purpose of improving the model’s generalization capability and mitigating the impact of domain gap, so as to finally increase the cross-domain prediction accuracy. Most person search methods are based on the person images (or image sequences) that have already been detected from the surveillance scene, and only focus on the stage of person reID, while the quality of person detection in practical application has significant impacts to the re-identification performance. Researchers have considered the two stages of person detection and person reID into an integrated task framework and put forward open-scene-based person search. Xiao et al. utilized a deep neutral network to handle the problem of person detection and person reID, and put forward online sample matching loss function to improve the network scalability for new samples [25]. Zheng et al. thought that the task of person search in open scene should be divided into two stages, detection and reID, and discussed the impact of different person detection algorithms and different person feature extraction algorithms on the final person search performance [26]. Li et al. first used CNN for feature extraction for the target person, and then used the extracted feature to guide recurrent neural network to gradually reducing the attention area, so as to locate the target person with the highest similarity in the whole surveillance scene [27]. Stefan et al. proposed an end-to-end person search model based on CNN with an attention mechanism. The model extracted both global and local features and boosted attention layers that allow the extraction of discriminative feature representations [28]. Dong et al. proposed an instance guided proposal network for person search [29]. Different from previous methods, the network used the appearance information of queries, the local relationship between proposals, and the global relationship in the scene to learn the similarity between queries and proposals end-to-end. It can reduce the number of proposals inputted to person reID by maintaining high-similarity bounding boxes. Zhong et al. presented an align-to-part network for person search [30]. The network refined the detected bounding box to cover the estimated whole body area, from which the distinctive part features can be extracted and aligned. The aligned part features were used for person reID in a local feature matching process. In this process, the effective part features were selected for similarity calculation, while the part features in occluded or noisy areas were ignored. This design enhances the robustness of person search to practical challenges.

### 2.2. Text-Based Person Search

IBPS requires a given target person image in practical application. The target image is often difficult to obtain, and it is relatively easy to obtain the appearance description of the interested person. In response to this situation, Li et al. presented a natural language description-based person search to look for the interested person image according to the obtained appearance description [6]. The paper discussed the performance of different text and image frameworks in person search task, and proposed a Recurrent Neural Network with Gated Neural Attention (GNA-RNN) model to learn the correspondence between text words and image features. How to use person identity label to increase the accuracy of cross-modal search has also aroused the interest of research workers. Li et al. put forward a two-phase training architecture based on identity recognition. Both phases use CNN-LSTM network framework [31]. In the first phase, cross-modal cross entropy (CMCE) was implemented to reduce the distances among similar samples and increase the distances among different samples. In the second phase, the potential co-attention method was used for network model refining. Similarly, Zheng et al. put forward a method using CNN to learn both text features and image features, using classification loss function to pre-train distinguishing features, and then using ranking function to train features matching. The method achieved good person search performance [17]. Chen et al. proposed a text and image block matching method to catch the local similarity [32]. The method can calculate the affinity between images and words, and precisely catch the local matching details among images and texts. Yamaguchi et al. put forward an approach, which combined the approaches of spatiotemporal person detection together with cross-modal retrieval, and the outputs are video clips instead of person images [33]. Zhang et al. put forward the loss function of cross-modal projection matching (CMPM) and the loss function of cross-modal projection classification (CMPC) to learn joint feature of text and image [7]. CMPM transforms the scalar projection between the text and image features into the matching probability, and learns the cross-modal matching by minimizing the KL difference among estimated matching probability distribution and true value matching probability distribution. CMPC loss function was also used to further increase the differences of features for interclass samples and enhance the compactness of features for intra-class samples. Jing et al. presented a multi-granularity attention network with guidance by posture, so that the cross-modal matching can be improved using the multilevel correlation between a person description and the corresponding visual content [8]. Shah et al. proposed the deep reinforcement learning method and used natural language description to help the agent find the boundary box around the target person. The method can remove the background, only focus on the person, and locate the person by adjusting the size of boundary boxes, so as to complete the person search [34]. Dong et al. put forward an Attribute-Image Hierarchical Matching (AIHM) model performing attribute and image matching for person search at both the global category-level and local attribute-level to solve the issues of existing text-based person search methods [3]. Aggarwal et al. presented a hierarchical text-based person search method [35]. First, the class attributes were extracted automatically from the text description of training data. Then, the hierarchical structure of features were learned, so that the middle-level features are attribute driven, and the high-level features are identity preserving. The two-level hierarchical features are used to obtain the retrieval results.

## 3. Proposed Method

**Figure 2:**
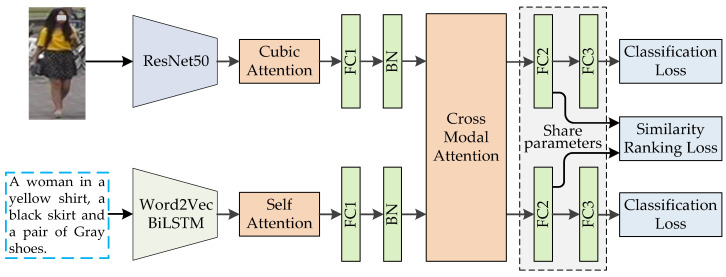
The architecture of the hybrid attention network for language-based person search. FC1, FC2, and FC3 denote full-connection layers, BN denotes batch-normalization layer.

Given a natural language description of the person, the goal of the task is to search for the closest person images from the large-scale image database. This task needs to deal with both CV and NLP, which is a fine-grained cross-modal retrieval issue. The architecture of the hybrid attention network we proposed is illustrated as Figure 2. The network is composed of three parts: (1) The image subnetwork, including a ResNet50 [36]-based CNN extended with a cubic attention mechanism, to extract the feature maps of person images. (2) The text subnetwork, including Word2Vec [37,38], the bidirectional LSTM (BiLSTM) network [13,14], and self-attention [15,16], to encode the natural language description and extract the semantic features. (3) The cross-modal joint learning module, including the cross-modal attention mechanism and full-connection layers (FC2 and FC3), with joint loss function (similarity ranking loss and classification loss), guiding the learning process to fully exploit the cross-modal and intra-modal information.

### 3.1. Image Feature Map Extraction

Because CNN achieved outstanding performance in many fields, this paper also uses CNN to extract person image visual features. In this paper, ResNet50 [36] with network parameters pre-trained on ImageNet [39] is adopted as base network to extract the features of person images. This paper designs a new cross-layer cubic attention mechanism. The mechanism enables the model to focus more on the key features of person images and ignore the redundant features, and leverages midlevel networks so that the model can focus more on the details of the image to improve the performance of this fine-grained task. The cubic attention mechanism is composed of two modules: One is the spatial attention module based on object region, and the other is the channel attention module based on object semantics. The details of each module are described below.

#### 3.1.1. Spatial Attention Based on Object Region

Different appearance attributes of the person described by natural language are distributed in different locations of the person images. In order to enable the model to focus more on the corresponding spatial location of person attribute objects, we improved the commonly used spatial attention model. This paper designs a spatial attention mechanism (SAM) based on the object region. The model can automatically acquire the locations and the detail information of the target objects in the image and weigh the spatial position so that the model is able to focus more on the important regions and restrain the impact of unimportant regions and noises.

In CNN, the lower-level features usually have higher resolution, can get more details, and are sensitive to small targets. Meanwhile, the higher-level features usually have lower resolution, contain more semantic information, and have larger receptive field, but may lack enough details and can easily ignore small objects. In the language description person search task, the appearance descriptions of the person (such as shoes, hand carry items, etc.) usually correspond to the small areas of person image, so detailed information is very important to achieve good performance of this fine-grained task. In order to get more detailed information, different from the other SAM-creating spatial attention map based on the last convolution layer (the conv5 layer of ResNet50), our SAM obtains spatial attention map based on the feature map of the midlevel of the network (conv4 layer of ResNet50). With the concern of the insufficient semantic information and high computational cost of low-level network, we did not create a spatial attention map based on the feature maps of lower layers, even though lower layers have higher resolution and more details.

**Figure 3:**
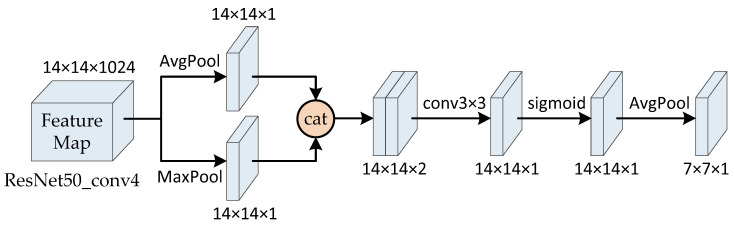
The structure of spatial attention mechanism. cat denotes catenation, conv3 × 3 denotes convolution operation with 3 × 3 kernels.

The structure of our SAM is illustrated as Figure 3. The channel dimension of the input feature map (extracted from conv4 layer of ResNet50) was handled with max pooling and average pooling, respectively, and two feature maps with channel dimension of 1 were obtained. Then, the two feature maps were concatenated to one feature map, and the feature map was reduced to one channel through the 3 × 3 convolution operation. Then, the attention map based on spatial location was generated using the sigmiod function. Then, the attention map was subsampled, and its height and width were half of the previous one. The final spatial attention feature map can be acquired through the multiplication of the spatial attention map and the output of channel attention feature map based on the conv5 layer of ResNet50. The calculation of spatial attention map is shown in Formula (1):

(1) $$M_{s}\left(F\right)=AvgPool(sigmoid\left(f^{3\times 3}\left(concat\left(AvgPool\left(F\right),MaxPool\left(F\right)\right)\right)\right))$$

where *F* is the input feature map, $M_{s}$ is the spatial attention map, and $f^{3\times 3}$ is the 3 × 3 convolution operation.

#### 3.1.2. Channel Attention Based on Object Semantics

Only spatial attention is not enough to achieve satisfactory performance, and it is necessary to obtain the features of person image from multiple dimensions. In the CNN model, the feature of each channel actually represents the weight of the image on different convolution kernels, and different features are dispersed in different channels. In order to get the semantic features of each channel obtain additional improvement on the performance of the task, we put forward a channel attention mechanism (CAM) based on object semantics.

**Figure 4:**
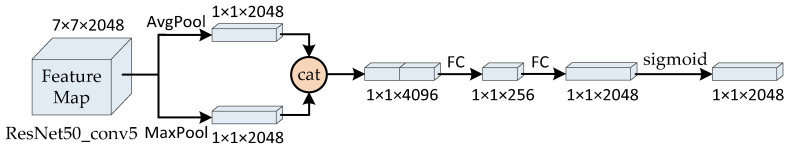
The structure of channel attention mechanism. FC denotes full connection, cat denotes catenation.

The structure of CAM is illustrated as Figure 4. The input feature map (extracted from the conv5 layer of ResNet50) was handled with max pooling and average pooling, respectively, to obtain two feature maps with height and width are 1. Then, the two feature maps were concatenated and connected with two layers of fully connected network, and then the attention map based on channel dimension was generated using the sigmiod function. The final channel attention feature map was generated through the multiplication of the channel attention map and the input feature map. The calculation of channel attention map is shown in Formula (2):

(2) $$M_{c}\left(F\right)=sigmoid\left(W_{2}\left(W_{1}\left(concat\left(AvgPool\left(F\right),MaxPool\left(F\right)\right)\right)\right)\right)$$

where *F* is the input feature map, $M_{c}$ is the channel attention map, and $W_{1}$ and $W_{2}$ are the weight matrix of the fully connected layer FC1 and FC2.

#### 3.1.3. Cross-Layer Cubic Attention

For CNN, lower layers features have higher resolution and more detail information than higher-layer features, and higher-layer features have more semantic information than lower layers. Spatial attention focuses on the details of objects, the features of the conv5 layer of ResNet50 were too abstract to provide sufficient detail and spatial information for an effective spatial attention to get achieve performance for such fine-grained tasks of the person search. So, different from other methods generating both SA and CA based on the conv5 layer of ResNet50, we proposed a cross-layer cubic attention mechanism which generates spatial attention based on the conv4 layer of ResNet50 and generates channel attention based on the conv5 layer of ResNet50. It can leverage both midlevel details and high-level semantics and achieve better performance of the fine-grained task.

**Figure 5:**
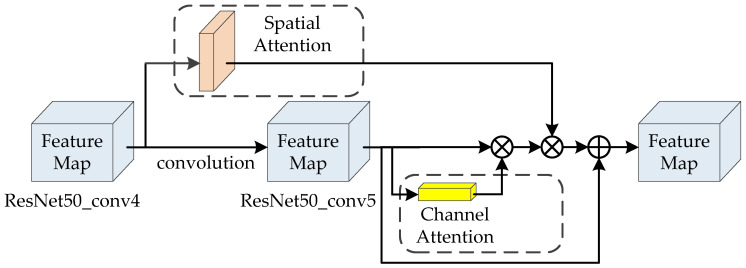
The structure of cubic attention mechanism. ⊗ denotes element-wise product, ⨁ denotes element-wise sum.

The structure of cross-layer cubic attention mechanism is shown in Figure 5. The cubic attention obtains important regions and key high-level semantic features of person image through SA and CA. SA and CA are combined to obtain the cubic attention weights of image features. With the purpose of preventing the disappearance of the gradient caused by the loss of feature information because of the multiplication of attention and features, we added a certain proportion of original features to the attention mechanism. The calculation of cubic attention feature map is shown in Formula (3):

(3) $$F_{att}=A_{c}⊗F_{i}⊗A_{s}+b*F_{i}$$

where $F_{i}$ is the output feature map of conv5 layer of ResNet50, $A_{s}$ is spatial attention, $A_{c}$ is channel attention, *b* is a hyper parameter, which is the weight for the proportion of original features of the conv5 layer of ResNet50 to prevent attention mechanism from introducing noise.

### 3.2. Natural Language Feature Extraction

**Figure 6:**
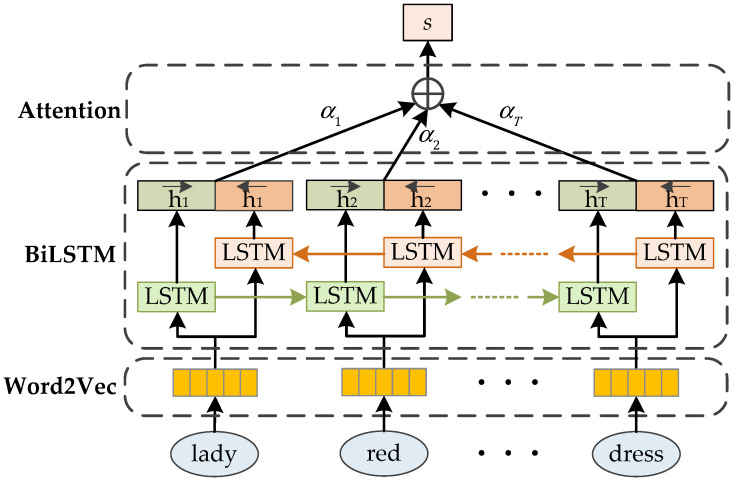
The text attention network structure for nature language feature extraction.

For the natural language description input of the person, we first used Word2Vec [37,38] to convert the words into the vectors containing semantic information. Then, we used BiLSTM [13,14] to acquire the context information and semantic features of the description. Finally, we used self-attention to set proper weights to different features in the description, and pay attention to discriminative features representing person appearance to effectively improve the person search efficiency and reduce the adverse effects of noises in the description. The text attention network structure of natural language extraction is illustrated as Figure 6.

#### 3.2.1. Word Embedding Using Word2Vec

Different witnesses have different language description methods for persons. Because there is no structured or standardized grammar and mode, the description is highly unstructured. Therefore, we cannot directly use the existing mathematical model or statistical model to process the description. We need to convert the words in the description into vectors for processing. Word2Vec [37,38], an open-source word embedding tool, uses the continuous bag of words (CBOW) or skip-gram model to transform words into high-dimensional real number vectors with certain semantic information. CBOW infers the central word through the context words. Skip-gram infers the context words through the central word. Comparing the two models, CBOW has shorter training time than skip-gram, but for some low-frequency words, the prediction effect of CBOW is poor, and its generalization capability is weak. Considering skip-gram has stronger generalization capability, this we selected Word2Vec’s skip-gram model for word embedding.

#### 3.2.2. Context Information Extraction Using BiLSTM

The descriptions of persons from different witnesses are in the form of natural language. Although the form is free, there is still a context dependent relationship in the description. The semantics can be understood more accurately according to the context information of the description. Recurrent neural network (RNN) can mine temporal and contextual semantic information of text, but with the increase of input, RNN’s perception of long-term information declines, resulting in the issues of long-term dependence and gradient disappearance [40]. The LSTM network [13] is an improvement for RNN and can solve the two issues mentioned above.

Although LSTM can solve the two main issues of RNN, LSTM can only learn the preceding information of the text and cannot use the following information of the text. However, in the language description of persons, the semantics of a word is closely relevant to both the preceding information and the following information. Therefore, using bidirectional LSTM (BiLSTM) [13,14] replaces LSTM and introduces the following information in the description, and can better capture bidirectional semantic dependency and improve the accuracy of prediction. BiLSTM is composed of two stacked LSTM networks, forward and backward, which capture the effective information of the preceding and the following, respectively, and then concatenate the two hidden states to form the final output. The BiLSTM network structure is shown in Figure 6.

For an input language description of person, such as a sentence {*w*_0_, *w*_1_,..., *w_t_*, *w_t+_*_1_,…}, the word vectors after word embedding are {*v*_0_, *v*_1_,…, *v_t_*, *v_t+_*_1_,…}, and BiLSTM can calculate the output $h_{t}$ of any time step, where *t* ∈ [1, 2, *t*,…, *T*]. ${\vec{h}}_{t}$ and ${\overset{\leftarrow }{h}}_{t}$ represent the outputs of forward LSTM and backward LSTM at time step *t*, respectively, as shown in Formula (4) and Formula (5), and the output of BiLSTM is determined by the state of LSTM networks in both directions, as shown in Formula (6).

(4) $$\vec{h_{t}}={\vec{LSTM}}_{t}\left(v_{1},v_{2},v_{3},\cdots ,v_{t}\right)$$

(5) $$\overset{\leftarrow }{h_{t}}={\overset{\leftarrow }{LSTM}}_{t}\left(v_{T},v_{T-1},v_{T-2},\cdots ,v_{t}\right)$$

(6) $$h_{t}=concat\left({\vec{h}}_{t},{\overset{\leftarrow }{h}}_{t}\right)$$

#### 3.2.3. Key Semantic Feature Extraction Using Self-Attention

In the description sentences of person, different words have different contribution to sentence semantics. In our work, we utilize self-attention in determining the contribution of every word and assigning different weights to it, so that the model can focus more on the critical features for this person search task, enhance the expression of key features, and weaken the influence of redundant features, which further improves the performance of person search based on natural language description.

The output of BiLSTM $h_{t}$ stands for the hidden vector for the *t*-th word, which contains the representation of context information. Different weights are assigned by self-attention to the output of BiLSTM at different time step; different weights represent different degrees of focus. The specific construction of attention is as follows.

First, the BiLSTM hidden layer $h_{t}$ is transformed into the hidden vector $u_{t}$ of the attention layer by the nonlinear tanh function, as shown in Formula (7):

(7) $$u_{t}=\tanh\left(w_{w}h_{t}+b_{w}\right)$$

where $w_{w}$ and $b_{w}$ are the coefficient matrix and bias vector of attention mechanism, which are updated automatically with model training.

Then, the attention weights $\alpha_{t}$ are acquired through normalization with the softmax function. $\alpha_{t}\in \left[0,1\right]$ represents the amount of semantic information of each hidden layer vector, and satisfies $\overset{T}{\underset{t=1}{\sum }}\alpha_{t}=1$, as shown in formula (8):

(8) $$\alpha_{t}=\frac{\exp\left(u_{t}\right)}{\sum_{t=1}^{T}\exp\left(u_{t}\right)}$$

Finally, weighted semantic vector is obtained by the weighted sum of hidden layer vector:

(9) $$s=\overset{T}{\underset{t=1}{\sum }}\alpha_{t}h_{t}$$

### 3.3. Cross-Modal Attention

Person search based on natural language description is a cross-modal retrieval task. The features of image and text are quite different. The task faces the challenge of cross-modal feature heterogeneity, and is easy to be disturbed by noises, with weak robustness. For the purpose of generating better cross-modal features and improving the performance, we put forward a cross-modal attention mechanism (CMAM), which can capture the correlation between text and image features, pay attention to the correlated parts between features, and improve the performance of this cross-modal task.

**Figure 7:**
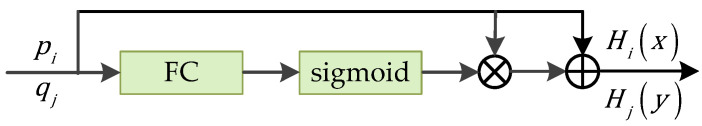
The structure of cross-modal attention mechanism (CMAM). $p_{i}$ and $q_{j}\;$ are the input features of CMAM for image subnetwork and text subnetwork, $H_{i}\left(x\right)$ and $H_{j}\left(y\right)\;$ are the output features respectively, FC denotes full connection layer, ⊗ denotes element-wise product, and ⨁ denotes element-wise sum.

As illustrated in Figure 2, after extracting the semantic feature of text and image, respectively, the two extracted features were inputted into the CMAM. The structure of CMAM is shown in Figure 7.

The CMAM utilizes a full connection layer followed with a sigmoid function to learn the weights of the input features. The parameters of CMAM are shared for image subnetwork and text subnetwork during feature weight learning, as it can cut down the number of parameters and create common feature expression space. The cross-modal attention map can be determined as follows:

(10) $$M_{i}\left(x\right)=sigmoid\left(W\left(p_{i}\right)\right)$$

(11) $$M_{j}\left(y\right)=sigmoid\left(W\left(q_{j}\right)\right)$$

where $p_{i}$ and $q_{j}\;$are the input features of CMAM for image and text, $M_{i}\left(x\right)$ and $M_{j}\left(y\right)$ are the cross-modal attention maps, and *W* is the weight matrix of the fully connected layer.

A fully connected layer and a nonlinear activation function can extract the whole semantic information of the feature map, capture the correlated parts of the cross-modal features, and ignore the irrelevant parts. The sigmoid function can map the weights of the learned features to [0, 1], and the attention mechanism can make the network to focus and extract relevant and important features. In addition, the mechanism of weight sharing can map the features of the image and text into a common feature expression space, which also shows that the features of one modality can be associated with those of another modality. The residual mapping adds the features in the original modal space, so that the feature weights learned are related to both the original features and the common features. The common feature space can better solve the problem of cross-modal heterogeneity.

After obtaining the feature weights, this paper combines the feature weights with the image and text features to obtain the final cross-modal feature representation. If just simply dot product features weight with features, the feature value may be too small. In addition, the ReLU [41] function in the network may aggravate the problem of feature sparsity, which may cause overfitting and weaken the robustness of the network. For the purpose of alleviating above issues, we added identity mapping in the mechanism. The final feature generation formulas of image and text are as follows:

(12) $$H_{i}\left(x\right)=\left(1+M_{i}\left(x\right)\right)\cdot p_{i}$$

(13) $$H_{j}\left(y\right)=\left(1+M_{j}\left(y\right)\right)\cdot q_{j}$$

where $H_{i}\left(x\right)$ and $H_{j}\left(y\right)\;$are the output feature maps of image and text. They are intermediate features, which will be input to the fully connected layer to obtain the final features.

The output of CMAM is connected to FC2 and FC3 for cross-modal joint learning as illustrated in Figure 2. Both the input and output dimensions of FC2 are 2048. The input dimension of FC3 is 2048, and the output dimension equals to number of person class which is the quantity of person IDs in person image dataset. Because cross-modal data have the characteristics that low-level features are heterogeneous but high-level semantic features are correlated, for this work, FC1 in the image subnetwork and FC1 in the text subnetwork adopt different parameter settings, while FC2 and FC3 in the image subnetwork and text subnetwork share the same parameters. This parameter setting strategy is not only beneficial to extract the effective modal specific feature representation of different modalities in the low-level layers, but also beneficial to establish the cross-modal semantic correlation in the high-level layers.

### 3.4. Joint Loss Function

As for loss function design, this paper proposes a new ranking loss embedded with intra-modal similarity, which can not only distinguish the positive and negative sample pairs coarsely, but also distinguish the similarity between the negative sample pairs in a fine-grained way, so as to obtain a more accurate ranking of the person based on the description. Additionally, we introduced the classification loss and use the identity label information to mine the intra-modal semantic information, so that the samples in the same modality belonging to the same person can be aggregated as much as possible to acquire more distinctive features. The classification loss was also utilized to restrain the similarity ranking loss during model training. Finally, this paper uses similarity ranking loss and classification loss to form joint loss function and jointly train the model to further improve the performance of natural language description based person search.

#### 3.4.1. Similarity Ranking Loss

In cross-modal tasks, ranking loss is widely used. The purpose of ranking loss is to make the distances among positive pairs shorter than that among negative pairs with a margin. It only focuses on cross-modal distance. This leads to two potential disadvantages: One is ignoring the intra-modal feature distribution, while the other is ignoring the feature similarity among samples in the same modality. A new ranking loss embedded with intra-modal similarity (similarity ranking loss) was proposed for this work. The similarity ranking loss not only considers the intermodal distance, but also considers the similarity among the different samples within the same modality and its influence on the final ranking results.

Ranking loss is to rank all person image samples in the dataset according to the distance of similarity with the language description of the person to be searched. For this image-text matching work, we used the Euclidean distance $D\left(g_{i},g_{t}\right)=\|g_{i}-g_{t}\|{}_{2}\;$as the measure of similarity between two samples. Here, $g_{i}$ and $g_{t}\;$ represent the sematic feature output by FC2 layer for the image sample *i* and text sample *t* respectively, and $\|\cdot {\|}_{2}$ represents $L_{2}$ norm. The ranking loss formula [42,43] is as follows:

(14) $$L_{rank}=\underset{T_{n}}{\sum }\underset{}{\max}\left[0,\alpha -D\left(g_{I_{p}},g_{T_{p}}\right)+D\left(g_{I_{p}},g_{T_{n}}\right)\right]\;+\underset{I_{n}}{\sum }\underset{}{\max}\left[0,\alpha -D\left(g_{T_{p}},g_{I_{p}}\right)+D\left(g_{T_{p}},g_{I_{n}}\right)\right]$$

Here, g represents the semantic features of image and text, α represents the margin parameter. *I* stands for image inputs, and *T* stands for text inputs. The first part of loss function is to sum up all mismatched text data $T_{n}$ given an image query $I_{p}$, and the second part is to sum up all mismatched image data $I_{n}$ given a text query $T_{p}$. The purpose of ranking loss is to make the distances among matched pairs of image-text samples (positive pair) shorter than that among any pair of mismatched image-text samples (negative pair). Since language-based person search uses a text query to retrieve images, only the second part of the ranking loss formula was calculated. During the actual training procedure, for the purpose of improving the calculation efficiency, ranking loss did not calculate and sum all the negative samples within the training datasets according to the above formula. We chose mini-batch to determine the sum of negative samples to consider both the efficiency of the calculation and the accuracy of the person search.

There is a disadvantage of the above ranking loss. For the language person search, its goal is not only to accurately match the samples to be searched, but also to meet the ranking rules, ranking according to the similarity relationship between all samples in the sample gallery and the samples to be searched. The above formula only maximizes the distances among positive and negative samples, which is coarse-grained. A novel similarity ranking loss is presented here to match the image and text features for such fine-grained cross-modal retrieval task of natural language description based person search, as shown in Formula (15):

(15) $$L_{srank}=\underset{T_{n}}{\sum }\underset{}{\max}\left[0,\alpha -D\left(g_{I_{p}},g_{T_{p}}\right)+\left(\beta -S\left(g_{I_{p}},g_{I_{n}}\right)\right)*D\left(g_{I_{p}},g_{T_{n}}\right)\right]\;+\underset{I_{n}}{\sum }\underset{}{\max}\left[0,\alpha -D\left(g_{T_{p}},g_{I_{p}}\right)+\left(\beta -S\left(g_{I_{p}},g_{I_{n}}\right)\right)*D\left(g_{T_{p}},g_{I_{n}}\right)\right]$$

We defined the intra-modal similarity $S\left(g_{a},g_{b}\right)$ to measure the similarity relationship of different samples in the same modality. Cosine distance was utilized here for intra-modal similarity evaluation. In the similarity ranking loss, we constrained the distance difference between the pairs of positive and negative samples. Assume β=1, and the value range of intra-modal similarity distance measured by the Cosine distance is [−1,1]. When $S\left(g_{I_{p}},g_{I_{n}}\right)\;$equals 1, which implies the positive and negative samples are identical, the loss function becomes $\underset{I_{n}}{\sum }\underset{}{\max}\left[0,\alpha -D\left(g_{T_{p}},g_{I_{p}}\right)\right]$, which only involves the distance of the pair of positive samples. In other words, during network training, if the images selected as negative samples are very similar to those of positive samples, it is not necessary to make the distance between positive and negative sample pair large enough. When $S\left(g_{I_{p}},g_{I_{n}}\right)\;$equals −1, which means that the negative sample is completely different from the positive sample, and the loss function becomes $\underset{I_{n}}{\sum }\underset{}{\max}\left[0,\alpha -D\left(g_{T_{p}},g_{I_{p}}\right)+2*D\left(g_{T_{p}},g_{I_{n}}\right)\right]$, so the loss function gives a greater weight to the distance between negative sample pairs, that is, the model is required to distinguish the distances among negative sample pairs and positive sample pairs as much as possible.

#### 3.4.2. Classification Loss

Similarity ranking loss mainly focuses on cross-modal correlation and similarity distance and cannot well explore the intra-modal distribution of features. In this paper, classification loss is introduced, and the intra-modal semantic information was extracted using the identity label information of the sample. Classification loss can constrain the similarity ranking loss based on identity labels, so that samples belonging to the same person identity within the same modality can be gathered together as much as possible, so as to obtain more distinctive features to further increase the accuracy of this task.

The identities (IDs) of different persons in the dataset were used as classes for this work. The IDs were used to classify images and language descriptions separately, and samples with the same ID in the same modality were classified into the same class. A full connection layer FC3 was added after the semantic features of image and text subnetworks. The output dimension of FC3 was the same as the quantity of person IDs (i.e., the quantity of classes) in the datasets. Then, softmax was used to calculate the probability of the class that each image and natural language description belongs to. Since person search can be treated as a multiclass classification task, cross-entropy loss was utilized to predict the identity. The classification losses of the person image and language description are expressed as following:

(16) $$p_{i}=softmax\left(z_{i}\right)$$

(17) $$L_{i}=-\overset{N}{\underset{k=1}{\sum }}y_{k\log p_{i}}$$

(18) $$p_{t}=softmax\left(z_{t}\right)$$

(19) $$L_{t}=-\overset{N}{\underset{k=1}{\sum }}y_{k\log p_{t}}$$

(20) $$L_{cls}=L_{i}+L_{t}$$

where $z_{i}$ and $z_{t}\;$represent the sematic feature output by FC3 layer for the image sample *i* and text sample *t*, respectively, $p_{i}\;and\;p_{t}\;$are the predicted probabilities, $y_{k}$ is the ground truth probability, $L_{i}$ and $L_{t}$ are the classification loss of the two modalities respectively, and $L_{cls}$ is the final classification loss joining both modalities.

The final loss function of joint cross-modal learning is as follows:

(21) $$Loss=\gamma_{1}L_{srank}+\gamma_{2}L_{cls}$$

where $\gamma_{1}$ and $\gamma_{2}\;$represent the weights of the losses.

## 4. Experiments and Discussions

### 4.1. Dataset and Evaluation Metric

Dataset. The CUHK-PEDES [6] dataset was utilized in the experiments, which is the only publicly available language-based person search dataset. It has 13,003 persons with 40,206 images in total, and each image has two language descriptions with a total of 80,412 descriptions. Most descriptions contain 20 to 40 words, with an average sentence length of 23.5 words. The training set consisted of 11,003 persons, 34,054 images, and 68,108 descriptions. The verification set consisted of 1000 persons, 3078 images, and 6156 descriptions. The test set included 1000 persons, 3074 images, and 6148 descriptions.

Evaluation Metric. We use top-1, top-5, and top-10 accuracy for performance evaluation. Provided by a language description, all images were sorted on the basis of the similarity to that description. The search was successful in the case that the relevant person was included in the first *k* images.

### 4.2. Implementation Details

The Tensorflow open-source machine learning platform was adopted in implementing the model for this work, with the following hardware environment: Intel i7-8700K CPU, NVIDIA GeForce RTX 2080Ti GPU, and 32-GB memory.

When learning the entire network of the cross-modal person search, a two-stage training strategy was adopted for the work. During the first stage, the classification loss was first adopted in the training of image subnetwork and text subnetwork separately, so that a language description or an image belonged to its corresponding identity class ($\gamma_{1}$ = 0, $\gamma_{2}$ = 1). The first stage of training can distinguish similar images and texts belonging to different identities in the same modality, and can learn the distinguishing features of different samples in the same modality. The separate learning of the two modalities based on classification loss in the first stage not only avoids cross-modal learning at the beginning, which may cause the entire learning process to become oscillating or divergent, but also provides a good initialization for the second stage of training. It is very helpful in accelerating the entire learning process convergence and improving the learning performance. In the second stage, the final joint loss function based on both classification loss and similarity ranking loss was utilized on the entire model to conduct cross-modal collaborative training on the samples ($\gamma_{1}$ = 1, $\gamma_{2}$ = 1). The model weights of the whole network, including image subnetwork, text subnetwork, and cross-modal learning module, were adjusted, and training was stopped when the training error tended to converge or reached the set epoch.

The ResNet50 of the image subnetwork uses the model weights that have been pre-trained using ImageNet, while the Word2Vec of text subnetwork uses the skip-gram model that has been pre-trained based upon Google News datasets with 300 dimensional vectors. The dimensions of the BiLSTM hidden layer and self-attention were set to 1024. The person images were resized to 224 × 224 before being input into ResNet50, and random horizontal flip data augmentation was used. The activation function of ReLU [41] was used between full-connection layers. The initial learning rate was configured as 0.001 in the first stage and 0.0002 in the second stage and decreased to one-tenth after the loss becomes stabilized. The SGD with momentum optimizer was adopted for both stages, with the momentum set to 0.5 for the first stage and 0.9 for the second stage. The batch sizes for both stages were 64. The first stage epoch was set to 80, the second stage epoch was set to 60.

### 4.3. Comparison with SOTA Methods

We compared our approach with following nine state-of-the-art (SOTA) approaches: CNN-RNN [44], NeuralTalk [45], GNA-RNN [6], Latent Co-attention [31], PWM + ATH [32], GLA [46], Dual Path [17], CMPM + CMPC [7], and PMA [8].

From the comparison result in Table 1, we can see our approach achieved the highest result (60.73% on top-1, 78.63% on top-5, and 84.96% on top-10) compared to other SOTA approaches. The following underlying reasons can be drawn out by deep analysis: (1) The cubic attention mechanism for feature extraction of image can pay more attention to critical parts and ignore unimportant or noise parts, and it can also make full use of both mid-layer detailed features and high-layer semantic features, which can extract better discriminative fine-grained feature representation than other methods. (2) BiLSTM can acquire the semantics of both the preceding and the following words and can better learn the bidirectional semantic dependency. Self-attention can pay more attention to the key words and ignore redundant or unimportant words. It can extract the context information and discriminative semantic features of the language description more effectively and accurately. (3) Cross-modal attention mechanism enables the network to extract correlated and important features between image and text and can better capture the cross-modal correlation and ignore uncorrelated information. (4) Similarity ranking loss can not only distinguish the positive and negative sample pairs coarsely, but also distinguish the similarity between the negative sample pairs in a fine-grained way, which can obtain a more accurate ranking of person images according to the description. (5) Classification loss can leverage the identity label information to mine the intra-modal semantic information, so as to acquire more distinctive features and make the samples belonging to the same person be aggregated as much as possible. (6) Similarity ranking loss and classification loss are complementary, as the joint loss function can better exploit the cross-modal and intra-modal correlation. The two-stage training can accelerate the convergence of the entire training procedure and improve the final performance.

In summary, the experiment result shows that our approach can acquire better performance for such fine-grained cross-modal nature language person search tasks and demonstrates the superiority of our approach for language-based person search.

### 4.4. Ablation Studies

The effectiveness of the proposed hybrid attention network for language-based person search is verified in this section, it includes four aspects: The cubic attention mechanism, text attention network based on BiLSTM and self-attention, cross-modal attention mechanism, and joint loss function. The CUHK-PEDES [6] dataset was used for all the ablation studies.

#### 4.4.1. Analysis of Cubic Attention Mechanism

The cross-layer cubic attention mechanism we proposed for image subnetwork has two distinguishing features: (1) Using both spatial attention and channel attention instead of just using one of them; and (2) leveraging both midlevel and high-level network features other than most other methods just based on high-level network features. The effectiveness of cubic attention mechanism was analyzed based on following comparisons: (1) Features extracted based on the conv5 layer of ResNet50, without any attention mechanism (ResNet50); (2) Features extracted from the spatial attention mechanism based on the conv5 layer of ResNet50 (ResNet50 + SA); (3) Features extracted from channel attention mechanism based on the conv5 layer of ResNet50 (ResNet50 + CA); (4) Features extracted from attention mechanism with both spatial attention and channel attention generated based on the conv5 layer of ResNet50 (ResNet50 + SA + CA); (4) Features extracted from cubic attention mechanism with spatial attention generated based on the conv4 layer of ResNet50 and channel attention generated based on the conv5 layer of ResNet50 (ResNet50 + cubic attention).

Based on the analysis on Table 2, it can be found that: (1) The methods having attention can obtain better results than those only based on the ResNet50 base network. It is because the attention mechanism can select the features that are more critical to the task from a large number of features, and put more weight on the selected features to obtain more detailed information of the target to be focused on while suppressing other useless features. (2) The methods based on both spatial attention and channel attention can obtain better performance than just using one of them. It is because the two attentions have a different focus. Spatial attention focuses on “where” the critical features are, while channel attention focuses on “what” the critical features are. The effects of the two attentions are complementary and combining them can achieve better performance. (3) The method based on cross-layer cubic attention can obtain better performance than the method generating both spatial and channel attentions on high-level of network (conv5 layer of ResNet50). It is because cubic attention generates spatial attention based on the conv4 layer of ResNet50 (midlevel) and generates channel attention based on the conv5 layer of ResNet50 (high-level), which can fully leverage the benefits of both the midlevel and high-level network. Spatial attention focuses on “where,” and it enables the network to focus more on the details of objects, and because the midlevel network has higher resolution and more detailed information than the high-level network, generating spatial attention based on the midlevel network can obtain better attention effect. Channel attention focuses on “what,” and it enables the network to obtain more effective feature information based on the channel, which means it focuses more on semantics. Because the high-level network has more semantic information than the midlevel, generating channel attention based on the high-level network can obtain better attention effect. The low-level network cannot provide sufficient semantics and the computation cost is high, so we chose to generate spatial attention based on the conv4 layer of ResNet50 instead of the lower layers.

**Figure 8:**
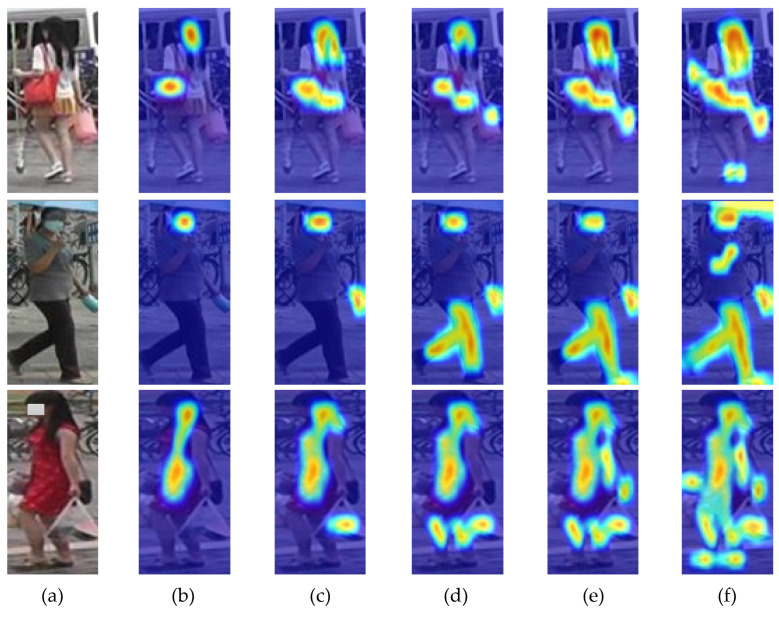
Examples of heat maps of different mechanisms. (**a**) Original person image; (**b**) ResNet50 without attention mechanism; (**c**) ResNet50 + SA; (**d**) ResNet50 + CA; (**e**) ResNet50 + SA + CA; (**f**) ResNet50 + cubic attention.

Figure 8 shows some examples of heat maps of the different mechanisms compared in this section. From the figure, we can see the cubic attention mechanism can focus more on the parts of the person images with discriminant features, locate the target regions more accurately, cover the target regions more completely, and have a higher overall weight and activation on the whole target regions, which is more conducive to the task.

In summary, the proposed cross-layer cubic attention mechanism is effective to extract discriminative feature and can achieve higher performance for such fine-grained tasks of language-based person search.

#### 4.4.2. Analysis of Text Attention Network

The text attention network we proposed includes three parts: Word2Vec, BiLSTM, and self-attention. The effects of BiLSTM and self-attention are analyzed in the section. The comparisons include: (1) LSTM without attention (LSTM), (2) BiLSTM without attention (BiLSTM), (3) LSTM with attention (LSTM + attention), and (4) BiLSTM with attention (BiLSTM + attention).

From Table 3, we can see: (1) The approaches based on BiLSTM can obtain better performance than the methods based on LSTM, with or without the attention mechanism. It is because LSTM can only learn the preceding information of the text, and cannot use the following information of the text. However, the semantics of a word is relevant to both the preceding information and the following information. BiLSTM can better capture the hidden dependency of context information and bidirectional semantic dependency, so as to extract richer semantic feature representation of the text description. (2) The approaches having attention obtained higher performance than the approaches having no attention. It is because the attention mechanism can consider the contribution of different words to semantics, increase the weight of key words, and reduce the weight of redundant or unimportant words, so as to obtain more discriminative feature representation of language description and make improvement on the performance. The analysis shows that the text attention network we proposed is effective in the language-based person search task.

#### 4.4.3. Analysis of Cross-Modal Attention Mechanism

For the purpose of verifying the effectiveness of the cross-modal attention mechanism (CMAM) proposed for the work, in the image subnetwork and text subnetwork, the cross-modal attention mechanism was replaced with a fully connected layer with the same number of channels. Other network structures remained unchanged, and the training method remained unchanged. Table 4 shows the performance based on the cross-modal attention mechanism was better than that based on the fully connected layer, the performance increases were 3.21% on top-1, 2.65% on top-5, and 2.09% on top-10, respectively. It demonstrates that the cross-modal attention mechanism can better capture the correlation between image and text, and it is more conducive to the cross-modal fine-grained task.

#### 4.4.4. Analysis of Joint Loss Functions

Different loss function has different constraint on the training of the model. This section explores the impacts of different loss functions on language-based person search performance and analyzes the effectiveness of the joint loss function we proposed. For the purpose of evaluating the similarity ranking loss, we proposed a comparison with commonly used ranking loss and included ranking loss in the comparison. The comparison includes the following five cases: Only ranking loss, only similarity ranking loss, only classification, ranking loss + classification loss, and similarity ranking loss + classification loss

By analyzing the experimental results in Table 5, the following conclusions can be drawn: (1) Only using a single loss cannot obtain a good result, and the methods of joining two losses can clearly improve the performance. (2) The performance of similarity ranking loss was higher than ranking loss, and the similarity ranking loss + classification loss is higher than ranking loss + classification loss, demonstrating the ranking loss embedded with similarity introduces similarity information between image samples in the process of network training, which can more accurately constrain the similarity distance between language description and image and improve the performance of language-based person search. (3) The experiment result demonstrates clearly the performance was effectively increased through fortifying with the classification loss based on the person identity label information. This proves that the identity label information can play a positive role in cross-modal person search. The reason is that other cross-modal loss functions focus on mining intermodal correlation while ignoring the intra-modal semantic correlation. The classification loss focuses on the feature distribution within the modal. After adding classification loss as a constraint guide for ranking loss or similarity ranking loss, the modal data of the same semantics will be gathered as much as possible. From the analysis, it can be seen that a joint loss function based on similarity ranking loss and classification loss can make additional improvement on the performance of this cross-modal fine-grained task of person search based on language description.

### 4.5. Qualitative Results

**Figure 9:**
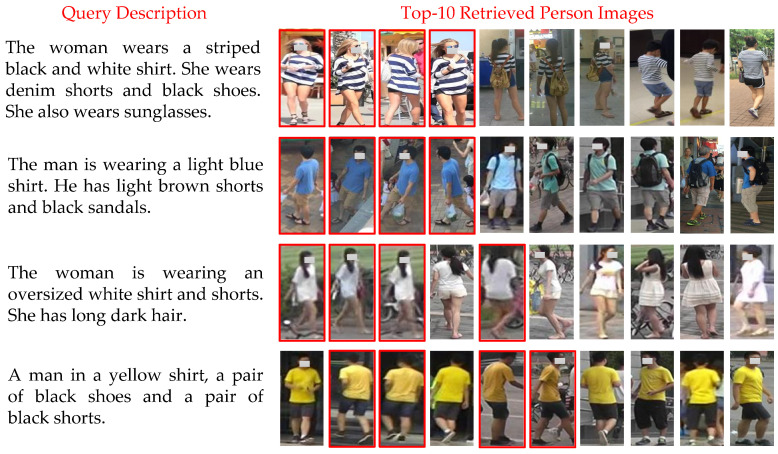
Examples of top-10 person search results by our method. The true matches are marked by red boxes.

Figure 9 illustrates some examples of language-based person search results using our approach on the CUHK-PEDES [6] dataset, where the red boxes indicate that the correct person images were searched. The first three rows in the figure show that our method can accurately learn the cross-modal correlation between the key appearance features described in the language descriptions and the images, and can search for the correct person images according to the appearance language descriptions. The result proves the effectiveness of the approach. The last row exposes an example of top-1 failure due to the high similarity of other person’s images and the description. However, it can be observed that the true matching images are still in the top positions in search ranking, which shows that our method has good robustness.

## 5. Conclusions

To improve the performance of the fine-grained cross-modal retrieval task of language-based person search, a novel hybrid attention network was proposed. The main novelties include: (1) A cross-layer cubic attention mechanism to extract the discriminative fine-grained feature map for person images. (2) BiLSTM and self-attention to encode the language description and extract effective semantic features. (3) A cross-modal learning module with cross-modal attention mechanism and joint loss function to fully exploit cross-modal and intra-modal information. The experiment result demonstrates that our approach acquires higher performance than other existing approaches and proves the efficiency and superiority of the approach. In future work, the approach will be applied to other general text-image datasets for further verification and improvement, and model compression and model distillation will also be introduced to improve the efficiency.

## Figures and Tables

**Table 1:** Comparison with state-of-the-art (SOTA) methods.

Method	Top-1	Top-5	Top-10
CNN-RNN [44]	8.07	-	32.47
NeuralTalk [45]	13.66	-	41.72
GNA-RNN [6]	19.05	-	53.64
Latent Co-attention [31]	25.94	-	60.48
PWM-ATH [32]	27.14	49.45	61.02
GLA [46]	43.58	66.93	76.26
Dual Path [17]	44.40	66.26	75.07
CMPM + CMPC [7]	49.37	-	79.27
PMA [8]	53.81	73.54	81.23
Ours	60.73	78.63	84.96

**Table 2:** Cubic attention mechanism effectiveness experimental results.

Method	Top-1	Top-5	Top-10
ResNet50	49.85	70.43	79.52
ResNet50 + SA	52.87	72.95	81.13
ResNet50 + CA	53.97	73.89	81.65
ResNet50 + SA + CA	57.64	76.48	83.37
ResNet50 + cubic attention	60.73	78.63	84.96

**Table 3:** Text attention network effectiveness experimental results.

Method	Top-1	Top-5	Top-10
LSTM	55.48	75.25	82.36
BiLSTM	57.56	76.47	83.37
LSTM + attention	58.66	77.42	83.94
BiLSTM + attention	60.73	78.63	84.96

**Table 4:** Cross-modal attention mechanism effectiveness experimental results.

Method	Top-1	Top-5	Top-10
full-connection layer	57.52	75.98	82.87
cross-modal attention mechanism	60.73	78.63	84.96

**Table 5:** Joint loss function effectiveness experimental results.

Method	Top-1	Top-5	Top-10
ranking	52.82	72.98	81.08
similarity ranking	54.85	74.45	82.11
classification	55.17	74.68	82.29
ranking + classification	58.72	77.15	83.92
similarity ranking + classification	60.73	78.63	84.96

## References

[1] Ye M., Shen J., Lin G., Xiang T., Shao L., Hoi S. (2020). Deep learning for person re-identification: A survey and outlook. arXiv.

[2] Wu A., Zheng W.-S., Guo X., Lai J.-H. (2019). Distilled person re-identification: Towards a more scalable system. Proceedings of the 2019 IEEE/CVF Conference on Computer Vision and Pattern Recognition (CVPR).

[3] Dong Q., Zhu X., Gong S. (2019). Person search by text attribute query as zero-shot learning. Proceedings of the 2019 IEEE/CVF International Conference on Computer Vision (ICCV).

[4] Lin Y., Zheng L., Zheng Z., Wu Y., Hu Z., Yan C., Yang Y. (2019). Improving person re-identification by attribute and identity learning. Pattern Recognit..

[5] Li Y., Xu H., Bian M., Xiao J. (2020). Attention based CNN-ConvLSTM for Pedestrian attribute recognition. Sensors.

[6] Li S., Xiao T., Li H., Zhou B., Yue D., Wang X. (2017). Person search with natural language description. Proceedings of the 2017 IEEE Conference on Computer Vision and Pattern Recognition (CVPR).

[7] Zhang Y., Lu H. (2018). Deep Cross-Modal Projection Learning for Image-Text Matching. Proceedings of the Haptics: Science, Technology, Applications.

[8] Jing Y., Si C., Wang J., Wang W., Wang L., Tan T. (2020). Pose-Guided Multi-Granularity Attention Network for Text-Based Person Search. Proceedings of the AAAI Conference on Artificial Intelligence.

[9] Corbetta M., Shulman G.L. (2002). Control of goal-directed and stimulus-driven attention in the brain. Nat. Rev. Neurosci..

[10] Larochelle H., Hinton G.E. Learning to combine foveal glimpses with a third-order Boltzmann machine. Proceedings of the 24th International Conference on Neural Information Processing Systems (NIPS).

[11] Zeiler M.D., Fergus R. Visualizing and Understanding Convolutional Networks. Proceedings of the European Conference on Computer Vision (ECCV).

[12] Woo S., Park J., Lee J.-Y., Kweon I.S. (2018). CBAM: Convolutional Block Attention Module. Proceedings of the Haptics: Science, Technology.

[13] Hochreiter S., Schmidhuber J. (1997). Long short-term memory. Neural Comput..

[14] Schuster M., Paliwal K. (1997). Bidirectional recurrent neural networks. IEEE Trans. Signal Process..

[15] Vaswani A., Shazeer N., Parmar N., Uszkoreit J., Jones L., Gomez A.N., Kaiser L., Polosukhin I. Attention is all you need. Proceedings of the 24th International Conference on Neural Information Processing Systems (NIPS).

[16] Bahdanau D., Cho K., Bengio Y. (2014). Neural machine translation by jointly learning to align and translate. arXiv.

[17] Zheng Z., Zheng L., Garrett M., Yang Y., Xu M., Shen Y.-D. (2020). Dual-path convolutional image-text embeddings with instance loss. ACM Trans. Multimedia Comput. Commun. Appl..

[18] Varior R.R., Shuai B., Lu J., Xu D., Wang G. (2016). A siamese long short-term memory architecture for human re-identification. Proceedings of the Computer Vision—ECCV 2016.

[19] Liu X., Zhao H., Tian M., Sheng L., Shao J., Yi S., Yan J., Wang X. Hydraplus-net: Attentive deep features for pedestrian analysis. Proceedings of the IEEE International Conference on Computer Vision (ICCV).

[20] He L., Liang J., Li H., Sun Z. (2018). Deep spatial feature reconstruction for partial person re-identification: Alignment-free approach. Proceedings of the 2018 IEEE/CVF Conference on Computer Vision and Pattern Recognition.

[21] Song C., Huang Y., Ouyang W., Wang L. (2018). Mask-guided contrastive attention model for person re-identification. Proceedings of the 2018 IEEE/CVF Conference on Computer Vision and Pattern Recognition.

[22] Guo Y., Cheung N.-M. (2018). Efficient and deep person re-identification using multi-level similarity. Proceedings of the 2018 IEEE/CVF Conference on Computer Vision and Pattern Recognition.

[23] Xin X., Wu X., Wang Y., Wang J. (2019). Deep Self-Paced Learning for Semi-Supervised Person Re-Identification Using Multi-View Self-Paced Clustering. Proceedings of the 2019 IEEE International Conference on Image Processing (ICIP).

[24] Fan H., Zheng L., Yan C., Yang Y. (2018). Unsupervised person re-identification. ACM Trans. Multimedia Comput. Commun. Appl..

[25] Xiao T., Li S., Wang B., Lin L., Wang X. (2017). Joint detection and identification feature learning for person search. Proceedings of the 2017 IEEE Conference on Computer Vision and Pattern Recognition (CVPR).

[26] Zheng L., Zhang H., Sun S., Chandraker M., Yang Y., Tian Q. (2017). Person re-identification in the wild. Proceedings of the 2017 IEEE Conference on Computer Vision and Pattern Recognition (CVPR).

[27] Li Y., Xu H., Bian M. (2019). End to end person re-identification based on attention mechanism. Proceedings of the IOP Conference Series: Materials Science and Engineering.

[28] Stefan L.-D., Abdulamit S., Dogariu M., Constantin M.G., Ionescu B. (2020). Deep learning-based person search with visual attention embedding. Proceedings of the 2020 13th International Conference on Communications (COMM).

[29] Dong W., Zhang Z., Song C., Tan T. (2020). Instance guided proposal network for person search. Proceedings of the 2020 IEEE/CVF Conference on Computer Vision and Pattern Recognition (CVPR).

[30] Zhong Y., Wang X., Zhang S. (2020). Robust partial matching for person search in the wild. Proceedings of the 2020 IEEE/CVF Conference on Computer Vision and Pattern Recognition (CVPR).

[31] Li S., Xiao T., Li H., Yang W., Wang X. (2017). Identity-aware textual-visual matching with latent co-attention. Proceedings of the 2017 IEEE International Conference on Computer Vision (ICCV).

[32] Chen T., Xu C., Luo J. (2018). Improving text-based person search by spatial matching and adaptive threshold. Proceedings of the 2018 IEEE Winter Conference on Applications of Computer Vision (WACV).

[33] Yamaguchi M., Saito K., Ushiku Y., Harada T. (2017). Spatio-temporal person retrieval via natural language queries. Proceedings of the 2017 IEEE International Conference on Computer Vision (ICCV).

[34] Shah A., Vuong T. (2018). Natural Language Person Search Using Deep Reinforcement Learning. arXiv.

[35] Aggarwal S., Babu R.V., Chakraborty A. (2020). Text-based person search via attribute-aided matching. Proceedings of the 2020 IEEE Winter Conference on Applications of Computer Vision (WACV).

[36] He K., Zhang X., Ren S., Sun J. (2016). Deep Residual Learning for Image Recognition. Proceedings of the 2016 IEEE Conference on Computer Vision and Pattern Recognition (CVPR).

[37] Mikolov T., Sutskever I., Chen K., Corrado G., Dean J. Distributed Representations of Words and Phrases and their Compositionality. Proceedings of the 27th International Conference on Neural Information Processing Systems (NIPS).

[38] Mikolov T., Corrado G., Chen K., Dean J. Efficient estimation of word representations in vector space. Proceedings of the International Conference on Learning Representations (ICLR).

[39] Deng J., Dong W., Socher R., Li L., Li K., Li F.F. ImageNet: A large-scale hierarchical image database. Proceedings of the IEEE Conference on Computer Vision and Pattern Recognition (CVPR).

[40] Bengio Y., Simard P., Frasconi P. (1994). Learning long-term dependencies with gradient descent is difficult. IEEE Trans. Neural Netw..

[41] Xu B., Wang N., Chen T., Li M. (2015). Empirical evaluation of rectified activations in convolutional network. arXiv.

[42] Karpathy A., Joulin A., Li F. Deep fragment embeddings for bidirectional image sentence mapping. Proceedings of the 28th International Conference on Neural Information Processing Systems (NIPS).

[43] Nam H., Ha J.-W., Kim J. (2017). Dual attention networks for multimodal reasoning and matching. Proceedings of the 2017 IEEE Conference on Computer Vision and Pattern Recognition (CVPR).

[44] Reed S., Akata Z., Lee H., Schiele B. (2016). Learning deep representations of fine-grained visual descriptions. Proceedings of the 2016 IEEE Conference on Computer Vision and Pattern Recognition (CVPR).

[45] Vinyals O., Toshev A., Bengio S., Erhan D. (2015). Show and tell: A neural image caption generator. Proceedings of the 2015 IEEE Conference on Computer Vision and Pattern Recognition (CVPR).

[46] Chen D., Li H., Liu X., Shen Y., Shao J., Yuan Z., Wang X. (2018). Improving deep visual representation for person re-identification by global and local image-language association. Proceedings of the Haptics: Science, Technology, Applications.

